# Single-port, via a midline approach, Robotic Transaxillary Thyroidectomy (SMART-Thyroidectomy): description of the technique and evaluation of feasibility in the first case series

**DOI:** 10.1007/s11701-026-03960-y

**Published:** 2026-09-18

**Authors:** Marco Raffaelli, Pierpaolo Gallucci, Ilda Hoxhaj, Francesco Pennestrì

**Affiliations:** 1 UOC di Chirurgia Endocrina e Metabolica, Fondazione Policlinico Universitario Agostino Gemelli IRCCS, Rome, 00168 Italy; 2 Centro di Ricerca in Chirurgia delle Ghiandole Endocrine e dell’Obesità (C.R.E.O.), Università Cattolica del Sacro Cuore, Rome, Italy

**Keywords:** Single-port robotic thyroidectomy, Da Vinci SP, Transaxillary thyroidectomy, Remote-access thyroidectomy

## Abstract

The Da Vinci SP system has recently been applied to remote-access robotic thyroidectomy, enabling single-port (SP) surgery with enhanced instrument articulation. We evaluated the feasibility of a novel Single-incision, via a Midline Approach, Robotic Transaxillary Thyroidectomy (SMART-T), which combines the cosmetic advantages of a concealed axillary incision with the bilateral midline exposure provided by the bilateral axillo-breast approach (BABA). A prospective analysis was performed on an initial series of patients who underwent SMART-T between June and July 2026. Demographic, operative, pathological, and postoperative data were collected. Sixteen patients successfully underwent SMART-T without conversion. All patients underwent lobo-isthmusectomy (11 right-sided and 5 left-sided procedures). Mean operative time was 99.6 ± 17.5 min, with mean docking and console times of 5.1 ± 2.0 and 55.6 ± 17.7 min, respectively. Ipsilateral central neck dissection was completed in three patients. One patient experienced transient minimal vocal cord hypomobility that resolved within 7 days. No other complication occurred. Postoperative pain and analgesic requirements were minimal. Subjective voice and swallowing impairment were minimal and completely recovered by postoperative day 7. Cosmetic outcomes were rated as excellent by 87.5% of patients. The present study reports the first European clinical series of SP BABA-derived thyroid procedures. SMART-T is a feasible single-port robotic approach to thyroidectomy that provides a BABA-like midline operative view through a single axillary incision, with encouraging early operative, functional, and cosmetic outcomes that support further evaluation in larger studies with longer follow-up.

**Supplementary Information:**

The online version contains supplementary material available at https://doi.org/10.1007/s11701-026-03960-y.

## Introduction

The Da Vinci SP system (Intuitive Surgical, Sunnyvale, CA, US) has recently been applied to various robotic extracervical approaches to thyroidectomy [1, 2]. The application of single-port (SP) technology could represent a further step in remote-access thyroid surgery by reducing invasiveness, while the flexible three-dimensional endoscope and multi-jointed wristed instruments introduced through a single cannula may facilitate dissection in narrow cervical spaces [3].

Several SP approaches have been described. Head-access techniques include the single-port robotic retroauricular approach (RA-SP) [4, 5] and single-port transoral robotic thyroidectomy (SP-TORT) [6–8], mainly used by head and neck surgeons. Chest-access techniques include single-port transaxillary robotic thyroidectomy (SP-TART/START) [9–14], gas-insufflation one-step single-port transaxillary (GOSTA) thyroidectomy [15–17], and the single-port robotic areolar approach (SPRA) [18–21]. All reduce flap dissection compared with multiport procedures.

SP-TART [9–12] and START ([13–14]) replicate multiport transaxillary robotic thyroidectomy (TART). The patient’s arm is elevated as in multiport TART, and an external retractor (Chung’s retractor) is used to elevate the clavicular head of the ipsilateral sternocleidomastoid muscle and the strap muscles, thereby maintaining the working space. The two approaches differ in the creation of the subcutaneous flap over the pectoralis major muscle. In START, this step is performed robotically, although redocking is required to modify the position of the retractor [13]. Both approaches provide lateral access to the thyroid, allowing good exposure of the ipsilateral lobe, whereas contralateral lobe exposure can be more challenging. GOSTA provides the same lateral access, but the patient’s arms remain alongside the body. The working space is maintained by low-pressure CO2 insufflation rather than by an external retractor [15–17].

To overcome the limitations of the lateral approach in terms of bilateral thyroid exposure and to avoid the risks associated with arm elevation, SPRA was proposed as a single-port modification of the bilateral axillo-breast approach (BABA) [18]. The patient is positioned as for BABA, with both arms alongside the body. A 3.0-cm incision for the SP cannula is made in the right superior periareolar region, while an additional 8-mm trocar is positioned in the contralateral periareolar region in the initial series or in one axilla, usually the right, in subsequent series [18–21]. The initial flap is created using hydrodissection and blind dissection with a tunnelizer to allow cannula insertion. Subplatysmal flap dissection is then continued under endoscopic vision with low-pressure CO2 insufflation (8 mmHg) to expose the anterior neck from the clavicles to the hyoid bone before docking the SP system. SPRA then follows the standard procedure through an anterior midline approach. The additional 8-mm trocar is used by the bedside assistant.

To overcome the limitations of transaxillary approaches (SP-TART, START, and GOSTA), including arm elevation, double docking, and lateral vision, and those of the areolar approach (SPRA), including blind initial dissection, a relatively large areolar incision, and the need for an additional incision for bedside assistance, we introduced a new approach designed to combine the advantages of transaxillary and breast procedures, based on our previous experience with both multiport TART and BABA.

The aim was to combine an axillary incision with a midline approach without arm elevation, exploiting the potential of CO2 insufflation and the SP system to retain a lateral incision while achieving a BABA-like operative view. A very similar concept was independently described by a Korean centre, combining an axillary incision with a midline approach, albeit with some technical differences [22].

Accordingly, the present study was designed to describe the technique of Single-incision Midline Approach Robotic Transaxillary Thyroidectomy (SMART-T) and to report the early postoperative outcomes of the initial series.

## Methods

Data from all patients who underwent SMART-T between June and July 2026 were prospectively recorded in a specifically designed institutional database and included in the present study. Demographic parameters (age, sex, BMI, and ASA score) and clinical parameters (thyroid laboratory tests, maximum nodule diameter, estimated thyroid volume, and cytological diagnosis) were recorded. Outcome measures included operative time, postoperative complications, postoperative stay, and final histology. Postoperative pain was assessed using a visual analogue scale (VAS; range 1–10) 4 h after surgery, at discharge on postoperative day 1 (POD1), and on POD7. All patients received a standard dose of paracetamol (1000 mg) at the end of surgery. Additional analgesic requirements during hospitalisation and after discharge until POD7 were recorded. Cosmetic outcome was evaluated at 2 weeks after surgery using a four-point verbal response scale (VRS) [23]: excellent, good, fair, or poor. Subjective voice and swallowing outcomes were assessed preoperatively, at discharge, and 1 week postoperatively using specifically designed questionnaires, the Voice Impairment Score (VIS; range 0–40) and the Swallowing Impairment Score (SIS; range 0–24) [24–27].

*Aims of the study* – The primary aim of the study was to evaluate the feasibility of SMART-T. Feasibility was defined as successful completion of the planned thyroid procedure through the SMART-T approach without conversion to another robotic or endoscopic approach or to conventional surgery. The secondary aim was to report its early postoperative outcomes.

*Indications* – Overall, indications for SMART-T were as follows: patient preference to avoid a neck scar, thyroid nodule ≤ 4.0 cm, and estimated thyroid volume ≤ 35 mL. Toxic nodules (Plummer’s adenoma) and Bethesda IV nodules were considered preferred indications. Small differentiated thyroid carcinomas (clinical T1 [cT1], Bethesda V or VI) were potentially eligible in the absence of ultrasonographic signs of local infiltration and/or overt gross central neck lymph node involvement (suspicious central neck nodes > 1 cm). Tumours other than differentiated thyroid carcinoma, cT2 (> 2 cm) tumours, gross central neck involvement, and lateral neck node involvement were not considered eligible. Male sex and thyroiditis were not considered contraindications. Class II or III obesity was considered a contraindication to the procedure.

*Surgical procedure* – The procedure is performed under general anaesthesia with orotracheal intubation. The C2 Xplore^®^ system (Inomed Medizintechnik GmbH, Emmendingen, Germany) is used for intraoperative neuromonitoring (IONM) of the laryngeal nerves. The patient is placed in the conventional supine position with both arms alongside the body. A small pillow is positioned beneath the shoulders to provide slight neck hyperextension. The skin of the neck and chest is prepared in the conventional manner. Draping leaves exposed a rhomboid-shaped area including the chin, anterolateral shoulder, axilla, and breast bilaterally.

**Fig. 1:**
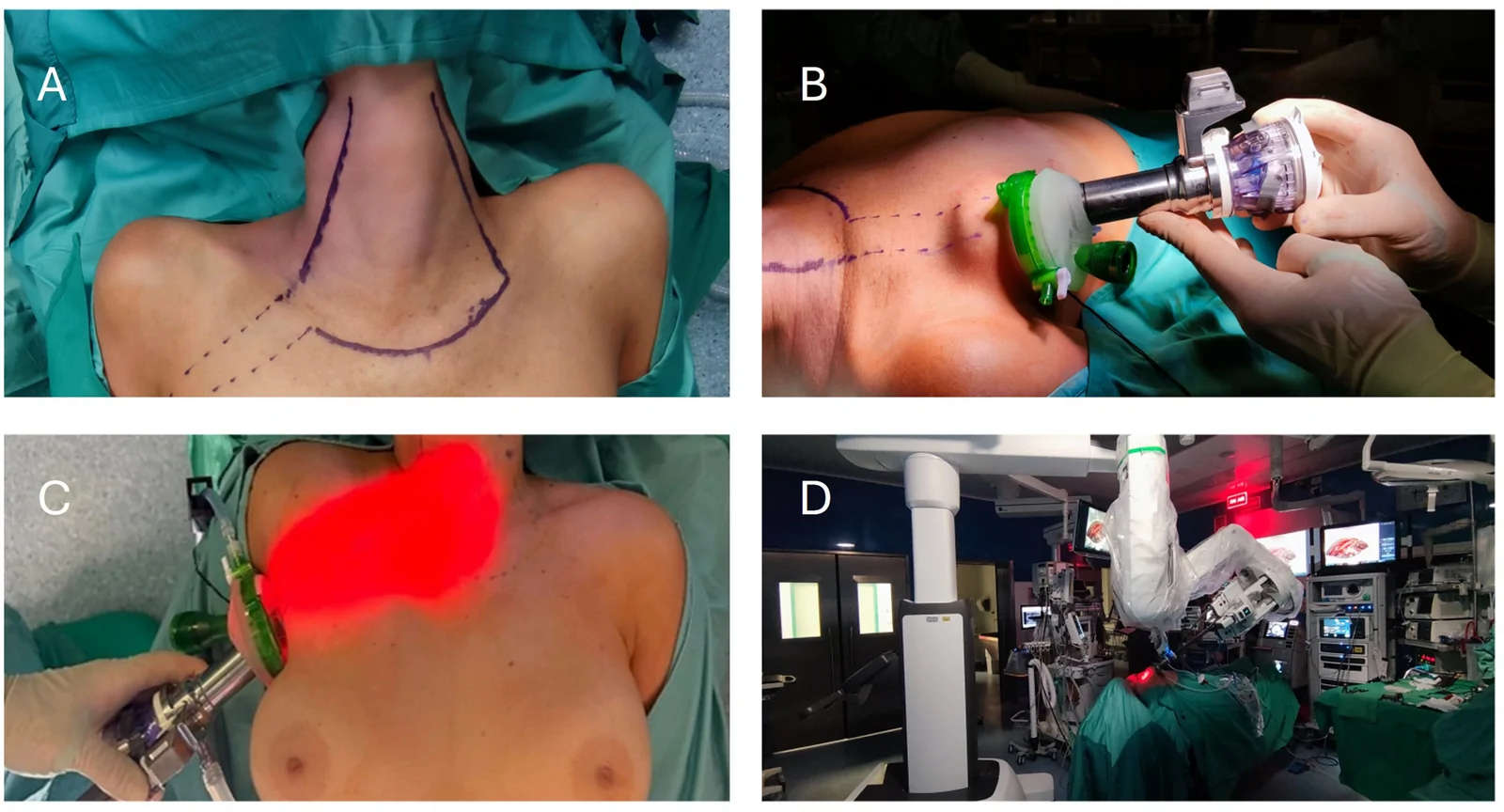
(**A**) Patient positioning and pre-operative markings. (**B**) Assembly of the GelPOINT™ Mini access system at the axillary incision, showing the SP 2.5-cm cannula and a 10-mm trocar positioned within the Gel Seal cap. (**C**) Area of subcutaneous flap before docking. (**D**) Docking setup of the da Vinci SP system

**Fig. 2:**
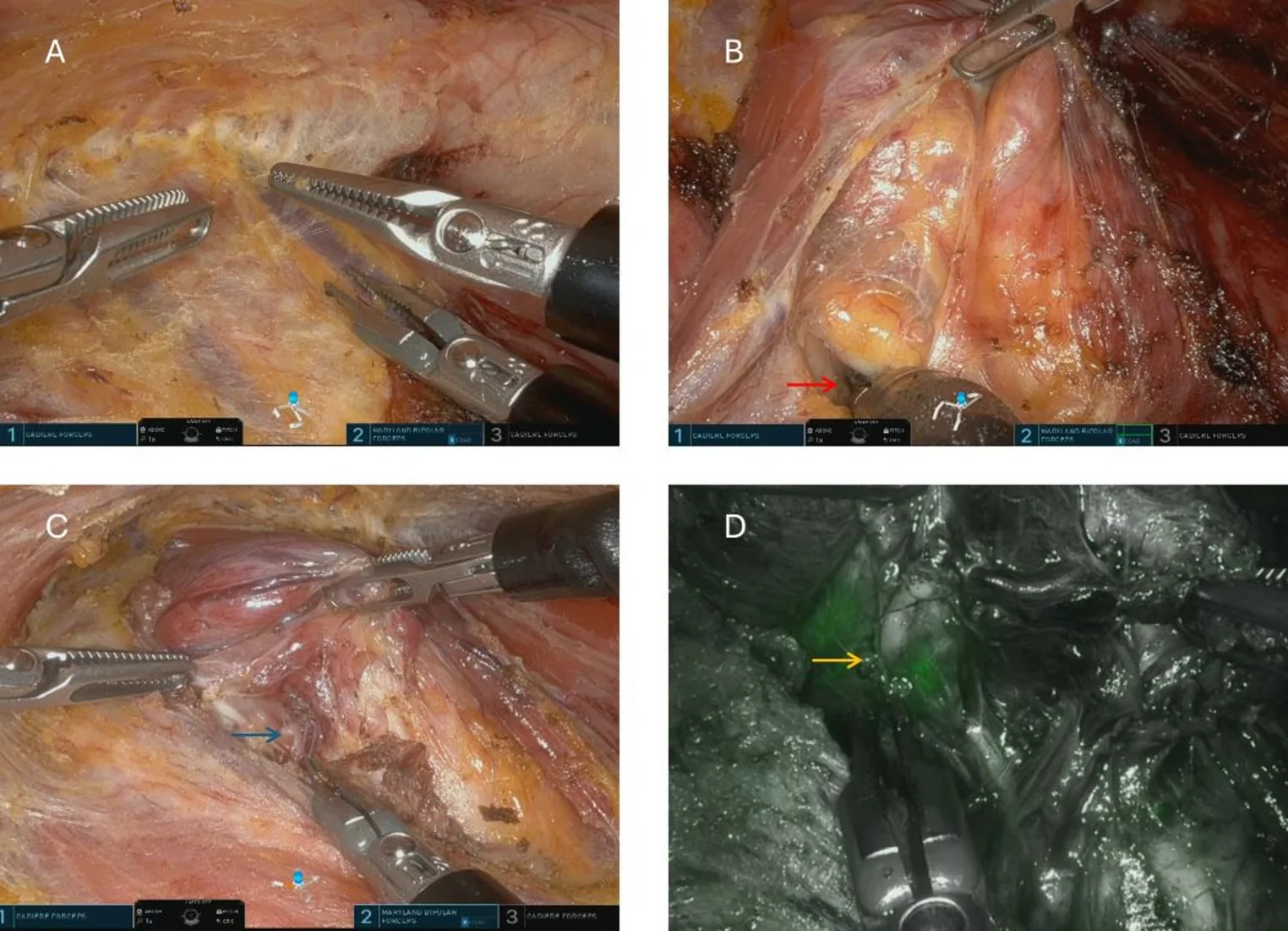
(**A**) Robotic view demonstrating instrument positioning, with the anterior cervical midline clearly identified as an anatomical landmark. (**B**) Exposure of the right neurovascular bundle with identification and stimulation of the right vagus nerve (red arrow), using Maryland forceps connected to the IONM system. (**C**) Medial retraction of the thyroid gland exposing the right inferior laryngeal nerve (blue arrow) that is stimulated using Maryland forceps connected to the IONM system. (**D**) ICG fluorescence imaging after administration of 1 mg indocyanine green, demonstrating vascularization of the inferior parathyroid gland before dissection (yellow arrow)

The markings on the patient’s skin in Fig. 1A indicate the planned area of subcutaneous dissection. A 3.0-cm skin incision is made along a natural skin crease in the right axilla, close to the superolateral margin of the right breast. A subcutaneous tunnel is then created under direct vision and external retraction along the anterior surface of the right pectoralis major muscle until the right clavicle is reached. At this point, the Alexis O Wound Protector-Retractor of the GelPOINT™ Mini access system (Applied Medical, Rancho Santa Margarita, CA 92688) is positioned at the axillary incision. The SP 2.5-cm cannula is inserted through the Gel Seal cap together with a 10-mm trocar (Fig. 1B). The cap is then secured to the wound protector, and CO2 insufflation is initiated at 8 mmHg. The fulcrum of the robotic trocar is positioned at the level of the skin incision, allowing elevation of the skin flap. Subcutaneous flap dissection is then continued under endoscopic vision using the SP flexible endoscope inserted through the entry guide and the LAP BiSect (Erbe Elektromedizin GmbH, Waldhoernlestrasse 17, 72072, Tuebingen, Germany) inserted through the accessory 10-mm port. The flap is extended to expose the anterior cervical region, including the anterior surfaces of the sternocleidomastoid and sternohyoid muscles bilaterally, from the hyoid bone to the clavicles (Figs. 1C and 2A).

The Da Vinci SP system is then docked. The robotic arm approaches from the head of the patient at an angle of 15–20° to the midline to provide BABA-like exposure. Once attached to the trocar, the robotic arm is lifted and medialized to retract the subcutaneous flap and obtain midline exposure (Fig. 1D).

The SP endoscope is positioned at 12 o’clock, two Cadiere forceps at 3 and 9 o’clock, and a Maryland bipolar forceps at 6 o’clock. Because of the midline exposure, no change in the internal configuration is required, and the right-sided access is used for both right- and left-sided thyroid procedures. Only bipolar cautery is used for dissection.

The operation begins with division of the strap muscles along the midline. The thyroid gland is exposed and progressively mobilised. The neurovascular bundle on the affected side is exposed, and the vagus nerve is stimulated using the Maryland forceps connected to the IONM system (Fig. 2B). The thyroid is then retracted medially, and the inferior laryngeal nerve (ILN) is exposed and stimulated (Fig. 2C). The inferior pole of the thyroid lobe is progressively mobilised, and the vessels are divided using bipolar cautery after identification and preservation of the inferior parathyroid gland (IPG). The isthmus is then divided close to the contralateral lobe using bipolar cautery. Isthmic dissection is continued cranially to expose both cricothyroid muscles and the thyroid cartilage.

Dissection continues at the superior pole of the thyroid lobe, where the vessels are divided using bipolar cautery and the external branch of the superior laryngeal nerve is exposed and stimulated. The lobe is then retracted medially using one of the two Cadiere forceps, and dissection of the ILN is continued to its entry into the larynx. The superior parathyroid gland (SPG) is usually identified close to the ILN entry point and preserved. Fluorescence imaging after administration of a fixed low dose (1 mg) of indocyanine green (ICG) is used to assess SPG vascularisation before its dissection, to facilitate preservation of a viable gland (Fig. 2D).

**Fig. 3:**
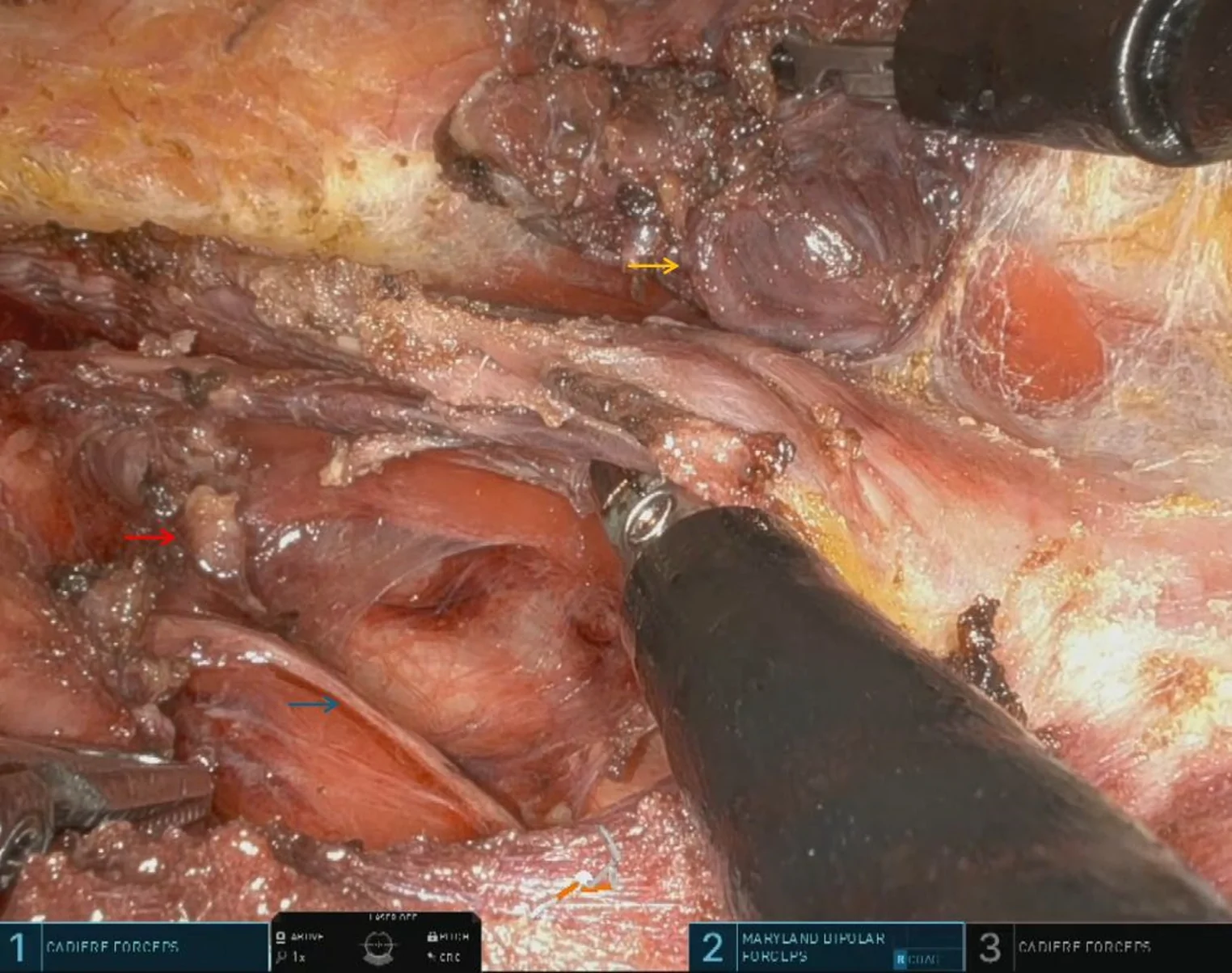
Operative field at the end of dissection (left lobo-isthmusectomy): the blue arrow indicates the left inferior laryngeal nerve, the red arrow indicates the superior parathyroid gland, and the yellow arrow indicates the thyroid specimen

Dissection is then continued along Berry’s ligament, and the thyroid lobe is detached. Haemostasis is checked using a Valsalva manoeuvre. The functional integrity of the ILN is assessed by direct and vagal stimulation. Definition and management of IONM loss of signal follow the standard institutional protocol ([28–29]). Vascularisation of the SPG and IPG is assessed by fluorescence imaging after a further administration of ICG (3 mg). Figure 3 shows the operative field at the end of dissection.

If total thyroidectomy is planned, the procedure is continued on the contralateral side.

The strap muscles are reapproximated along the midline using a running barbed suture.

The specimen is put in a plastic bag and removed.

**Fig. 4:**
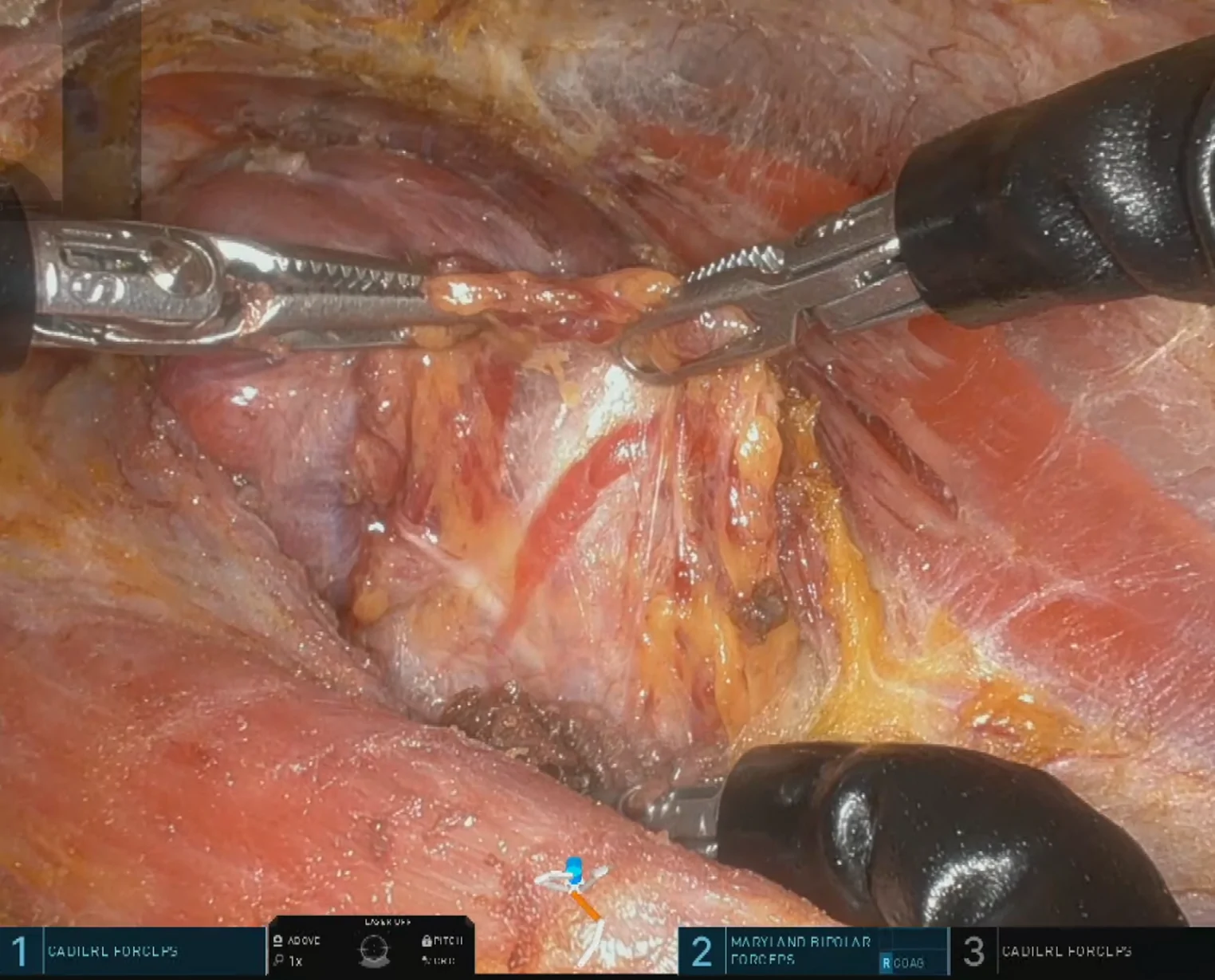
En bloc dissection of the ipsilateral central neck compartment (peri-recurrential, paratracheal, and pretracheal lymph nodes)

In patients with proven or suspected papillary thyroid carcinoma (Bethesda V and VI), ipsilateral central neck node dissection (peri-recurrential, paratracheal, and pretracheal nodes) is performed en bloc with the inferior pole of the thyroid lobe (Fig. 4). The ipsilateral central neck nodes are then retrieved in a plastic bag and sent for frozen section evaluation (FSE) before further dissection, in accordance with the institutional protocol [30–32]. Thyroid lobe dissection is continued while awaiting the FSE result. Contralateral dissection is performed if FSE demonstrates ipsilateral central neck node metastases [30–33].

After specimen removal, the SP system is de-docked. No drain is left inside. The wound is closed with a running suture.

A pressure dressing is positioned over the subcutaneous flap on the chest wall for at least 12 h.

The key steps of SMART-T are demonstrated in Supplementary Videos S1–S4: axillary incision and initial subcutaneous dissection (Video S1), endoscopic flap dissection (Video S2), docking of the Da Vinci SP system (Video S3), and the robotic thyroidectomy procedure (Video S4).

*Postoperative management* – As in conventional thyroid surgery, patients remain in the recovery room for 1 h after the procedure. They are encouraged to stand 4 h after returning to the ward and are allowed to drink clear liquids at the same time. Patients are discharged 24 h after surgery. In patients undergoing bilateral thyroid resection, PTH is measured 4 h after surgery and serum calcium levels on POD1, in accordance with the institutional protocol [34]. Postoperative laryngoscopy is performed by an ENT consultant on POD1 before discharge.

*Statistical analysis* – For descriptive analysis, continuous variables are reported as means (± standard deviation). Categorical variables are reported as frequency counts (n) and percentages (%). Exploratory CUSUM analyses of total operative time and console time were performed to assess the procedural learning curve.

## Results

Among 311 thyroid procedures performed between June and July 2026, 37 (11.9%) patients were selected for robotic thyroidectomy. Of these, 16 consenting patients (5.2% of all thyroid procedures) underwent SMART-T. The remaining 21 patients selected for robotic surgery underwent robotic BABA (6 lobectomies and 15 total thyroidectomies, with central neck dissection in 8 and lateral neck dissection in 2) because they either declined the new approach (*n* = 6) or did not meet the initial indications.

Patients’ characteristics are reported in Table 1. Preoperative diagnoses included a toxic nodule in 1 case, follicular neoplasm (Bethesda IV) in 12 cases, and a suspicious or malignant nodule of follicular origin (Bethesda V or VI) in the remaining 3 cases.

**Table 1:** Patients’ characteristics

Number of cases	16
**Sex** Male/Female (%)	1 (6.3%)/15 (93.7%)
**Mean age**, ± SD, [range], years	39.8 ± 10.6 [24–59]
**Mean BMI**, ± SD, [range], kg/m2	23.0 ± 2.2 [19.7–28.7]
**Preoperative diagnosis**	
**Toxic nodular goitre** **Bethesda IV** **Bethesda V/VI**	1 (6.3%) 12 (75%) 3 (18.7%)
**Mean lesion size**, ± SD, [range], mm	25.5 ± 10.1 [10–40]
**Procedure**	
**Loboisthmusectomy (LT)** **Side** Right/Left	16 (100%) 11 (68.8%) / 5 (31.2%)
**Ipsilateral central neck dissection**	3 (18.75%)
**Overall mean operative time**, ± SD, [range], min	99.6 ± 17.5, [73–148]
**Mean flap time**, ± SD, [range], min	21.0 ± 7.29, [14–39]
**Mean docking time**, ± SD, [range], min	5.06 ± 1.98, [3–9]
**Mean console time**, ± SD, [range], min	55.6 ± 17.7, [29–109]
**Complications (%)**	
**Transient vocal cord palsy** **Definitive vocal cord palsy** **Other complications**	1 (6.25%) 0 0
**Mean hospital stays**, ± SD, days	1.0 ± 0.0
**Mean VAS score, ± SD, [range]**	
4 h after surgery POD 1 POD 7	2.5 ± 1.1 [1–5] 1.0 ± 1.0 [ 0–4] 0.0 ± 0.0
**Mean dose of paracetamol administered, ± SD, [range], gram**	
Day of surgery POD 1 POD 7	1.5 ± 0.5 [1–2] 0.9 ± 0.6 [ 0–2] 0.0 ± 0.0
**Voice Impairment Score (VIS), SD, [range]**	
Pre-operative POD 1 POD 7	0.0 ± 0.0 0.69 ± 0.70 [0–2] 0.0 ± 0.0
**Swallowing Impairment Score (SIS), SD, [range]**	
Pre-operative POD 1 POD 7	0.0 ± 0.0 0.13 ± 0.50 [0–2] 0.0 ± 0.0
Early Cosmetic Outcome* (%)	
Excellent Good Fair Poor	14 (87.5%) 2 (12.5%) 0 0
**Pathological diagnosis**	
Benign NIFTP pT1am PTC pT1b PTC Median number of removed nodes (range) N0 / N1a	11 (68.75%) 1 (6.25%) 2 (12.5%) 2 (12.5%) 6 (4–8) 3 / 0

*Self-reported at POD15

Abbreviations: BMI- body mass index, VAS - Visual analog scale, POD – Postoperative Day, PTC- Papillary thyroid carcinoma, NIFTP - Non-invasive Follicular Thyroid Neoplasm with Papillary-like Nuclear Features

Mean preoperative nodule size was 25.5 ± 10.1 mm (range: 10–40).

Overall, 16 thyroid lobectomies were performed (11 right and 5 left). In the 3 patients with Bethesda V or VI cytology, ipsilateral central neck dissection was performed (2 right-sided and 1 left-sided). FSE of the removed nodes was negative in all 3 cases, and no further dissection was required.

Overall skin-to-skin operative time was 99.6 ± 17.5 min (range: 73–148). Mean flap creation time before docking was 21.0 ± 7.29 min (range: 14–39), mean docking time was 5.06 ± 1.98 min (range: 3–9), and mean console time was 55.6 ± 17.7 min (range: 29–109).

Type I (segmental) partial loss of signal (< 50%) on intraoperative neuromonitoring (IONM) was recorded in two patients at the end of dissection; however, POD1 laryngoscopy confirmed normal vocal cord motility in both. In one female patient, IONM showed normal post-dissection curves, whereas POD1 laryngoscopy demonstrated minimal asymptomatic ipsilateral vocal cord hypomotility (reduced abduction), which had resolved by POD7. No other complications occurred. In particular, no bleeding, seroma, chest wall hypoesthesia or paraesthesia, or skin burns were observed.

Postoperative stay was 1 day (24 h) in all patients, in accordance with the institutional protocol.

Mean VAS pain score was 2.5 ± 1.1 (range: 1–5) 4 h after surgery, 1.0 ± 1.0 (range: 0–4) on POD1, and 0 in all patients on POD7. The mean dose of paracetamol administered was 1.5 ± 0.5 g (range: 1–2) on the day of surgery, 0.9 ± 0.6 g (range: 0–2) on POD1, and 0 g in all patients on POD7.

No patient had preoperative voice or swallowing impairment (VIS and SIS = 0 in all patients at baseline). Minimal voice impairment was observed on POD1 (mean VIS 0.69 ± 0.70; range: 0–2) and had completely resolved 1 week after surgery (mean VIS 0). Only one patient had minimal swallowing impairment (SIS 2 on POD1), which had completely resolved 1 week after surgery. All other patients reported no swallowing impairment after surgery (SIS 0 on POD1 and POD7).

**Fig. 5:**
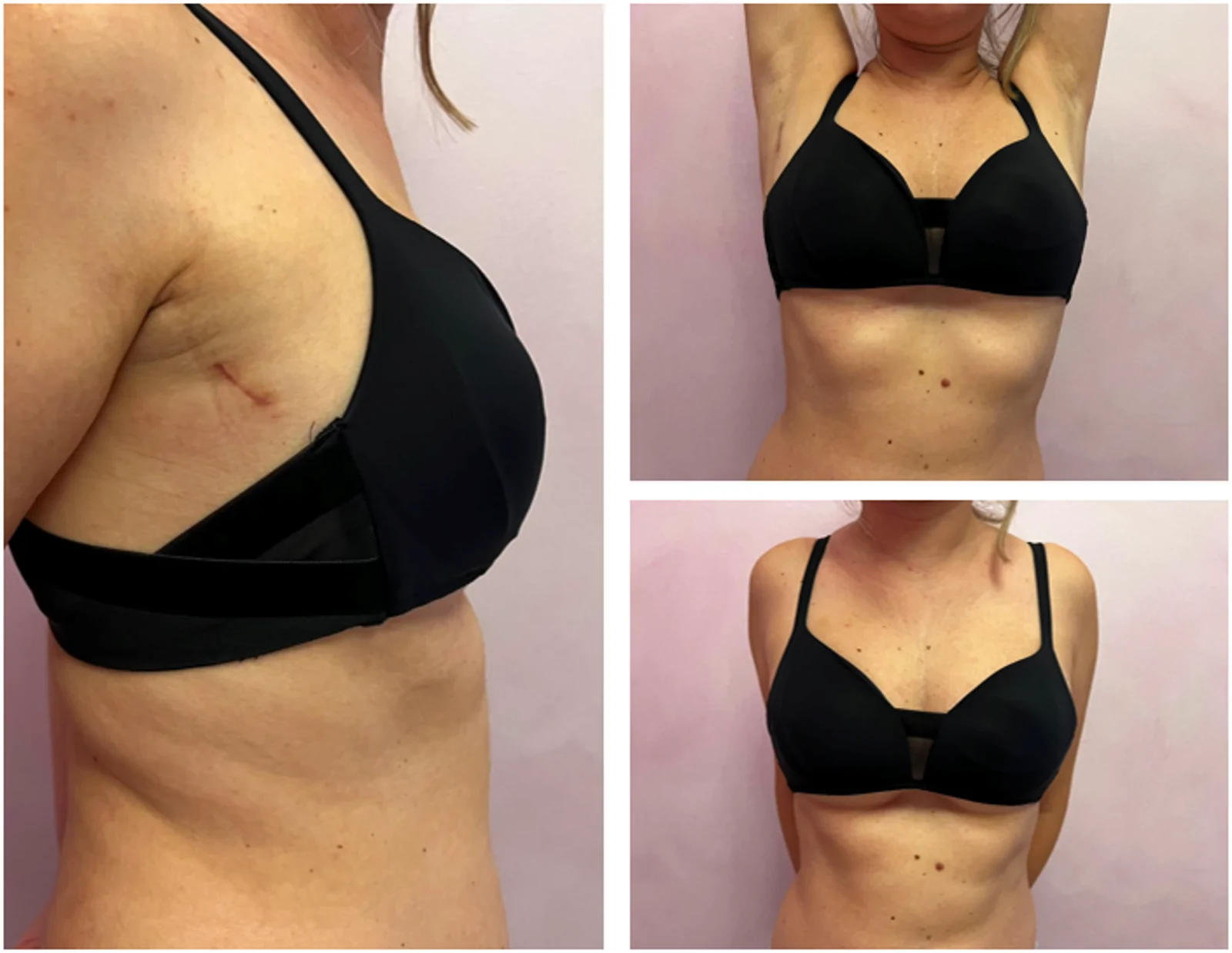
Cosmetic result on postoperative day 15

Cosmetic outcome was rated as excellent by most patients (14/16) at the first follow-up visit (POD15). Figure 5 shows the cosmetic result on POD15. Only two patients rated the cosmetic outcome as good.

Final histology showed benign disease in 11 patients, a Non-invasive Follicular Thyroid Neoplasm with Papillary-like Nuclear Features (NIFTP) in 1 case, 2 pT1b PTC and 2 incidental pT1am PTC.

In the three patients who underwent CND, the median number of removed ipsilateral central neck nodes was 6 (range 4–8). No patient showed lymph node metastasis at final histology.

After tumour board discussion, no patient with PTC was considered for completion thyroidectomy.

Learning curve – Total operative time decreased in the second half of the series compared with the first (mean 101.8 vs. 97.4 min). CUSUM curves showed a change in trajectory at case 12 (breakpoint), followed by relative stabilisation of operative performance (plateau phase). Ipsilateral central neck dissection was introduced at case 12; from case 13 onwards, operative performance remained relatively stable despite the increased procedural complexity (Supplementary Fig. 1).

## Discussion

This initial case series suggests that SMART-T is feasible and associated with low morbidity, relatively short operative time, and encouraging early cosmetic, voice, and swallowing outcomes.

Although independently developed and technically different, our approach yielded findings consistent with a recent report showing that a midline approach to SP thyroidectomy through an axillary incision is feasible and can reproduce the principal steps of BABA [22].

Soon after its introduction, the Da Vinci SP system was applied to remote-access thyroid procedures [4, 6, 9]. Four main approaches have since been described: transoral [6–8], retroauricular [4, 5], transaxillary [9–17], and areolar [18–21]. Supplementary Table 1 summarises the chest-access techniques.

Transaxillary approaches have received the greatest attention, mainly because of the early adoption of SP technology in Korean centres and the intrinsic limitations of multiport TART, in which multiple robotic arms must be introduced through a single skin incision with the aid of an external retractor (Chung’s or Modena retractor) [35]. The narrow transaxillary working space may result in robotic arm collisions when using multiport technology [12]. Published series suggest that SP technology may help overcome this limitation.

Recently published large cohorts from Korean centres have demonstrated the feasibility and safety of SP transaxillary approaches (SP-TART and START), including in patients with malignant disease and associated central or lateral neck lymph node dissection [12–14]. Nonetheless, in SP-TART and START, the arm on the side of the incision needs to be elevated to reduce the distance between the axilla and neck and to facilitate subcutaneous flap creation and positioning of the external retractor. Arm hyperextension may cause brachial plexus injury, a feared, although extremely rare, complication of transaxillary approaches [36].

To overcome this risk, an alternative approach without arm abduction or external retraction and using low-pressure CO2 insufflation has been proposed [15–17]. GOSTA represents a valid alternative to SP-TART and START and is associated with a reduced workload for the bedside assistant.

However, dissection between the two heads of the sternocleidomastoid muscle on the affected side provides a lateral view in all transaxillary approaches, which may limit exposure and dissection on the opposite side, although bilateral thyroid resection is feasible. Nonetheless, in the published series, contralateral lobe resection was performed in fewer than 10% of cases [12, 13, 15].

Bilateral exposure is better achieved through a midline approach, as in multiport BABA thyroidectomy. However, BABA requires a wide anterior chest wall flap, which may cause transient hypoesthesia or paraesthesia in up to 41% of patients [36]. SPRA reduces flap dissection while preserving craniocaudal view and bilateral access, and a recent large single-surgeon series confirmed its feasibility and safety, including for bilateral thyroid resection and lateral neck dissection [21]. Nonetheless, SPRA requires a 3.0-cm right areolar incision and an additional 8-mm port for initial flap creation and bedside assistance; the initial flap is also created blindly using a tunnelizer [21].

The Da Vinci SP system (Intuitive Surgical, Sunnyvale, CA, US) was introduced at our tertiary referral centre in May 2026 and, after appropriate company training, we initiated its use for robotic thyroid procedures.

When we introduced the SP platform, we aimed to exploit its potential (single cannula, flexible endoscope, and multi-jointed wristed instrumentation) while capitalising on the experience gained over the previous 7 years with TART and, more recently, robotic BABA. Ultimately, our aim was to obtain BABA-like exposure while avoiding the need for an additional port and an areolar incision.

We opted for a right axillary incision close to the superolateral margin of the breast, concealed within a natural skin crease and behind the anterior axillary line. We hypothesised that the SP robotic arm connected to the SP cannula could act as a subcutaneous flap retractor, allowing a craniocaudal medial view similar to that achieved with robotic BABA. To achieve this, we chose to use the conventional metallic SP cannula rather than the plastic SP Access Port Kit. To allow effective CO2 insufflation and maintain an adequate working space, the SP cannula was inserted through the cap of the GelPOINT™ Mini access system (Applied Medical, Rancho Santa Margarita, CA 92688), which also allows introduction of an additional assistant trocar and smoke evacuation during dissection. This configuration provided BABA-like exposure (Fig. 2A) while substantially reducing the extent of anterior chest wall dissection. Specifically, chest wall dissection was limited to a 3.0-cm-wide subcutaneous tunnel from the axillary incision to the right clavicle. Cervical dissection was then performed to the same extent as in BABA and conventional thyroid surgery, exposing the strap muscles from the hyoid bone to the clavicles.

Of note, the approach we developed is very similar to the recently published single-port axillary-to-midline (SAM) approach [22], which was reported after we had already started our own programme. This convergence highlights the need to find an appropriate compromise between axillary and areolar approaches, combining their advantages while avoiding their respective limitations. However, compared with SAM, the superior and medial retraction provided by the metallic SP cannula, together with the lower position of the incision, appears to provide SMART-T with exposure closer to that of BABA and SPRA. In the SAM series, the author suggested using an ipsilateral axillary incision to achieve adequate exposure, and contralateral resection was performed in fewer than 8% of cases (5/63). By contrast, in our series, contralateral (left) thyroid resection was achieved through the right axilla in 30% of cases (5/16), although no patient underwent total thyroidectomy. This potential advantage requires confirmation in patients undergoing bilateral thyroid resection.

Mean operative time in our series appeared shorter than those reported for the SP-TART, START, and SAM approaches and similar to that reported for SPRA, suggesting that the midline craniocaudal view may facilitate dissection even during the initial experience. However, these are indirect descriptive comparisons, and only formal prospective comparative studies can support definitive conclusions.

Only one patient, in the absence of any IONM alteration, experienced minimal asymptomatic vocal cord hypomotility, which completely resolved by POD7. No other complications occurred. In particular, no hypoesthesia or paraesthesia along the flap was observed, possibly reflecting the absence of blind dissection and the avoidance of a tunnelizer.

Postoperative pain and analgesic consumption were minimal. Moreover, subjective voice and swallowing impairment were minimal and had completely resolved in all patients by POD7. These findings appeared favourable when descriptively compared with other minimally invasive and robotic procedures [25, 37]; however, no definitive conclusions can be drawn in the absence of formal comparative analyses.

The cosmetic result was graded as excellent by most of the patients (87.5%).

Exploratory CUSUM analysis suggested early stabilisation of operative performance, with a change in trajectory at case 12 (breakpoint) followed by a plateau phase (Supplementary Fig. 1). Thereafter, operative performance remained relatively stable despite a progressive increase in procedural complexity (i.e. central neck dissection with frozen section examination of the central neck nodes), suggesting an early improvement in overall operative workflow.

The preliminary SMART-T findings are encouraging. Nonetheless, this was a very early experience in carefully selected patients, and the observed outcomes may have been influenced by patient selection.

Remote-access robotic thyroidectomy has so far been adopted predominantly in East Asian countries, particularly Korea and, to a lesser extent, China. This is especially true for SP procedures, as most published clinical series originate from Korean centres.

Of note, the present study represents the first European series of an SP BABA-derived procedure. To date, only a short report describing two European cases of SP-TART has been published [38].

With increasing interest in remote-access thyroid procedures [36], the introduction of the Da Vinci SP system may contribute to expanding the adoption of robotic thyroidectomy in Europe and other Western countries.

In conclusion, despite the limitations related to the small single-centre sample size, the highly selected patient population, the lack of a control group and bilateral thyroid resections, and the very short follow-up, the present series supports the feasibility of SMART-T and showed encouraging preliminary results in terms of operative time, complication rate, postoperative pain, early cosmetic outcome, and voice and swallowing outcomes. SMART-T may represent an alternative to other SP approaches for robotic thyroidectomy by combining features of transaxillary and breast approaches. In addition, this represents the first series of SP BABA-derived robotic thyroidectomy from a European country. Nonetheless, larger studies with longer follow-up are required to confirm these preliminary findings and determine the role of SMART-T in the surgical management of patients with thyroid disease.

## Supplementary Information

Below is the link to the electronic supplementary material.

Supplementary Material 1: Supplementary Video S1. SMART Thyroidectomy: incision and initial dissection. The video demonstrates the right axillary skin incision and creation of the initial subcutaneous tunnel along the anterior surface of the right pectoralis major muscle towards the right clavicle.

Supplementary Material 2: Supplementary Video S2. SMART Thyroidectomy: endoscopic flap dissection. The video demonstrates continuation of the subcutaneous flap under endoscopic vision, with exposure of the anterior cervical region and creation of the midline operative view.

Supplementary Material 3: Supplementary Video S3. SMART Thyroidectomy: docking. The video demonstrates docking of the Da Vinci SP system and positioning of the robotic arm to provide superior and medial retraction of the subcutaneous flap while maintaining the midline exposure.

Supplementary Material 4: Supplementary Video S4. SMART Thyroidectomy: robotic procedure. The video demonstrates the main steps of the robotic thyroidectomy through the midline approach using the Da Vinci SP system.

Supplementary Material 5

Supplementary Material 6

## Data Availability

The datasets generated and analysed during the current study are not publicly available because they contain potentially identifiable clinical information but are available from the corresponding author on reasonable request and subject to approval by the relevant institutional authorities.
