# Supplementary material for: Single-port, via a midline approach, Robotic Transaxillary Thyroidectomy (SMART-Thyroidectomy): description of the technique and evaluation of feasibility in the first case series
Source: J Robot Surg. 2026 Sep 18;20(1):1001. doi: 10.1007/s11701-026-03960-y (PMC13588920; doi:10.1007/s11701-026-03960-y)
Supplement: Supplementary file 1 [file 11701_2026_3960_MOESM5_ESM.docx]

**Supplementary Figure 1.** CUSUM analysis of total operative time and robotic console time across the first 16 SMART-T procedures: first 8 cases (phase I) versus second 8 cases (phase II), with a change in trajectory around case 12 and relative stabilisation thereafter.
