# Supplementary material for: Single-port, via a midline approach, Robotic Transaxillary Thyroidectomy (SMART-Thyroidectomy): description of the technique and evaluation of feasibility in the first case series
Source: J Robot Surg. 2026 Sep 18;20(1):1001. doi: 10.1007/s11701-026-03960-y (PMC13588920; doi:10.1007/s11701-026-03960-y)
Supplement: Supplementary file 2 [file 11701_2026_3960_MOESM6_ESM.docx]

**Supplementary Table 1.** Characteristics of SP robotic thyroidectomy approaches using chest access (transaxillary and areolar approaches)

| **Approach** | **Incision** | **Arm position** | **CO₂ insufflation** | **Entry system** | **External retractor** | **Additional incision** | **Docking** | **Access to the thyroid bed** | **View** | **Extent of subcutaneous flap dissection** | **Contralateral exposure** |
| --- | --- | --- | --- | --- | --- | --- | --- | --- | --- | --- | --- |
| SP-TART  (9) | Ipsilateral axillary | Hyperextended | No | SP cannula | Yes^2^ | No | Single | Between SCM heads and beneath strap muscles | Lateral | Axilla-to-ipsilateral SCM | Feasible^8^ |
| START  (13) | Ipsilateral axillary | Draped and extended^1^ | No | SP cannula | Yes^2^ | No | Two-step | Between SCM heads and beneath strap muscles | Lateral | Axilla-to-ipsilateral SCM^5^ | Feasible^8^ |
| GOSTA  (15) | Ipsilateral axillary | Alongside the body | Yes, 8 mmHg | SP Access Port Kit | No | No | Single | Between SCM heads and beneath strap muscles | Lateral | Axilla-to-ipsilateral SCM^6^ | Feasible^8^ |
| SPRA  (18-21) | Right periareolar | Alongside the body | Yes,  6-8 mmHg | SP cannula | No^3^ | Yes  8-mm trocar | Single | Midline | Cranio-caudal | Breast-to-neck^7^ | Adequate^9^ |
| SAM  (22) | Ipsilateral axillary | Alongside the body | Yes,  6 mmHg | SP Access Port Kit | No | No | Single | Midline | Lateral | Axilla-to-neck^7^ | Feasible^8^ |
| SMART-T  (current study) | Right axillary | Alongside the body | Yes,  8 mmHg | SP cannula | No^4^ | No | Single | Midline | Cranio-caudal | Axilla-to-neck^7^ | Adequate^9^ |

^1^ arm position changes during the second step of flap creation; ^2^ Chung’s or Modena retractor; ^3^ superior flap retraction with the SP cannula; ^4^ superior-medial flap retraction with the SP cannula; ^5^ partially performed robotically using the two-step method; ^6^ robotic dissection between the SCM heads and beneath the strap muscles; ^7^ anterior neck exposed from the clavicles to the hyoid bone; ^8^ ipsilateral access optimises ipsilateral exposure; ^9^ right-sided access used in all cases

**Abbreviations**: SP-TART = single-port transaxillary robotic thyroidectomy; START = single-port transaxillary robotic thyroidectomy with two-step retraction; GOSTA = gas-insufflation one-step single-port transaxillary; SPRA = single-port robotic areolar approach; SAM = single-port axillary to midline; SMART-T = Single-incision Midline Approach Robotic Transaxillary Thyroidectomy; CO₂ = carbon dioxide; SCM = sternocleidomastoid muscle.
